# The Warburg Effect in Endothelial Cells and its Potential as an Anti-angiogenic Target in Cancer

**DOI:** 10.3389/fcell.2018.00100

**Published:** 2018-09-11

**Authors:** Gillian Fitzgerald, Inés Soro-Arnaiz, Katrien De Bock

**Affiliations:** Laboratory of Exercise and Health, Department of Health Sciences and Technology, Swiss Federal Institute of Technology Zurich, Zurich, Switzerland

**Keywords:** angiogenesis, endothelial, metabolism, cancer, vascular normalization

## Abstract

Endothelial cells (ECs) make up the lining of our blood vessels and they ensure optimal nutrient and oxygen delivery to the parenchymal tissue. In response to oxygen and/or nutrient deprivation, ECs become activated and sprout into hypo-vascularized tissues forming new vascular networks in a process termed angiogenesis. New sprouts are led by migratory tip cells and extended through the proliferation of trailing stalk cells. Activated ECs rewire their metabolism to cope with the increased energetic and biosynthetic demands associated with migration and proliferation. Moreover, metabolic signaling pathways interact and integrate with angiogenic signaling events. These metabolic adaptations play essential roles in determining EC fate and function, and are perturbed during pathological angiogenesis, as occurs in cancer. The angiogenic switch, or the growth of new blood vessels into an expanding tumor, increases tumor growth and malignancy. Limiting tumor angiogenesis has therefore long been a goal for anticancer therapy but the traditional growth factor targeted anti-angiogenic treatments have met with limited success. In recent years however, it has become increasingly recognized that focusing on altered tumor EC metabolism provides an attractive alternative anti-angiogenic strategy. In this review, we will describe the EC metabolic signature and how changes in EC metabolism affect EC fate during physiological sprouting, as well as in the cancer setting. Then, we will discuss the potential of targeting EC metabolism as a promising approach to develop new anti-cancer therapies.

## Introduction

The circulatory system is an ingenious and extended network of blood and lymphatic vessels that allows the transport of nutrients and oxygen from their site of uptake or production to peripheral tissues where they will be metabolized. At the same time, it removes any metabolic waste products away from these tissues. Vessels are lined by an impressive monolayer of endothelial cells (ECs), the surface of which can cover > 700 m^2^ and has a weight of about 700g in an adult person (Wolinsky, 1980). Although in the adult organism ECs seldom proliferate and remain in a quiescent state for protracted periods, they retain the ability to rapidly initiate the formation of new vessels; a tightly coordinated process termed angiogenesis. The expansion of the vascular network via angiogenesis occurs in response to nutrient and oxygen deprivation; vascular expansion serves to accommodate these enhanced oxygen and nutrient requirements and to restore tissue metabolic homeostasis. Angiogenesis and metabolism are thus intimately linked. This is particularly true in a cancer setting, where the nutrient and oxygen requirements of tumors exceeding a volume of 1 mm^3^ surpass what can be provided through passive diffusion from the vessels of the surrounding host tissue. When this occurs, the tumor microenvironment starts releasing proangiogenic factors, such as the vascular endothelial growth factor (VEGF), fibroblast growth factor (FGF), ephrins, and angiopoietins, promoting the vascularization of the tumor and the restoration of oxygen and nutrient supply. This process, termed the angiogenic switch, is crucial for the growth and progression of the tumor. Anti-angiogenic therapy has therefore been put forward as an attractive therapeutic avenue for anti-cancer therapies (Folkman, 1971; Yuan et al., 1996). Therapeutically, anti-angiogenic treatment has been proposed to starve existing tumors of nutrients and oxygen preventing their continued growth. In recent years, the concept of angio-prevention has emerged as a prophylactic strategy to stop low grade undetected lesions from progressing by preemptively providing anti-angiogenic therapy to at risk patients (Albini et al., 2012). This preventative strategy complements the traditional therapeutic approach. Although several strategies to inhibit VEGF have been developed and approved for the treatment of cancer, they have shown only limited efficacy (Jayson et al., 2016; Fukumura et al., 2018). This is in part due to the upregulation of alternative pro-angiogenic growth factors within the tumor to overcome VEGF blockade (Bergers and Hanahan, 2008; Ellis and Hicklin, 2008; Carmeliet and Jain, 2011; Jayson et al., 2016; Fukumura et al., 2018). This has necessitated the development of novel treatment strategies that target not just the angiogenic growth factors but rather the endothelium itself. In recent years, it has become clear that ECs reprogram their metabolism during angiogenesis, and targeting endothelial metabolism provides a promising alternative therapeutic target in anti-cancer anti-angiogenic strategies (De Bock et al., 2013a; Cantelmo et al., 2017).

In this review, we will give a brief overview of angiogenic biology and the canonical signaling pathways involved in this process. For a more comprehensive overview of this topic, we refer the reader to the following reviews (Adams and Alitalo, 2007; Potente et al., 2011; Blanco and Gerhardt, 2013; Eelen et al., 2018). Although angiogenesis can occur via different mechanisms, endothelial metabolism has been exclusively investigated during the sprouting of new vessels out of existing ones (sprouting angiogenesis). We will therefore limit our review to sprouting angiogenesis, beginning with an overview of the Warburgian characteristics of ECs, and how they change their metabolism during angiogenesis. Then, we will highlight recent insights into the potential of targeting endothelial metabolism as a novel anti-angiogenic strategy for cancer therapy.

## Angiogenesis – the Current Model of Vessel Sprouting

Vessel growth via sprouting angiogenesis is initiated via the secretion of angiogenic growth factors from the oxygen and nutrient deprived microenvironment, which triggers tip cell selection. Tip cells are characterized by a migratory (non-proliferative) phenotype with numerous and highly motile filopodia which explore the microenvironment and guide the nascent sprout toward the hypoxic/nutrient deprived area (Gerhardt et al., 2003). Importantly, the tip cell subsequently instructs the neighboring cells not to become tip cells. Instead, those cells then adopt a stalk cell fate, characterized by a proliferative (non-migratory) phenotype which provides a mechanism for sprout extension (Hellstrom et al., 2007; Potente et al., 2011). Moreover, stalk cells drive the formation of the nascent vascular lumen (Iruela-Arispe and Davis, 2009; Charpentier and Conlon, 2014; Betz et al., 2016). When two tip cells make filopodial contacts, the sprouts eventually anastomose, a new blood vessel is formed, and blood flow is initiated (Lenard et al., 2013; Betz et al., 2016). After the functional vascular network has been established it remodels in order to optimize tissue perfusion and oxygen/nutrient delivery (Korn and Augustin, 2015; Ricard and Simons, 2015). Ultimately, the secretion of angiogenic growth factors will cease and this, together with blood flow, will instruct ECs to return to quiescence. Those quiescent ECs, termed phalanx cells, secrete a basement membrane, recruit pericytes, and form tight junctions via the upregulation of VE-Cadherin expression (Mazzone et al., 2009).

Although many other angiogenic growth factors have been described and characterized, VEGF is a key regulator of sprouting angiogenesis. Following release by hypoxic and nutrient deprived cells, it binds to the VEGF receptor 2 (VEGFR2) that is expressed by ECs, and initiates a signaling cascade that promotes EC migration, proliferation, and survival. At the same time, VEGF induced cytoskeletal dynamics activate a transcriptional program by promoting the activation of the transcriptional co-activators YAP and TAZ (Kim J. et al., 2017; Wang et al., 2017; Neto et al., 2018). YAP/TAZ control cytoskeletal rearrangements for filopodia formation and junctional dynamics; their nuclear translocation promotes EC proliferation. VEGF signaling in the tip cell also results in the upregulation of delta like 4 (DLL4), which binds the Notch1 receptor of the neighboring stalk cells and prevents them from acquiring tip cell characteristics (Suchting et al., 2007; Benedito et al., 2009). Also, Notch signaling lowers VEGFR2 levels and enhances the expression of the VEGF trap VEGFR1, rendering the stalk cell less responsive to VEGF. Cell fates within the growing sprout are transient and ECs continuously overtake each other, alternating at the tip cell position (Bentley et al., 2009; Jakobsson et al., 2010; Arima et al., 2011). ECs stochastically change their fate during sprouting as a consequence of cellular motion during sprouting angiogenesis (Boas and Merks, 2015) and the cell at the tip is constantly replaced, even in absence of VEGF (Arima et al., 2011; Boas and Merks, 2015). Subsequently, VEGF-DLL4-Notch signaling ensures that the cell that ended up at the tip position adopts the tip cell phenotype (Arima et al., 2011).

**FIGURE 1:**
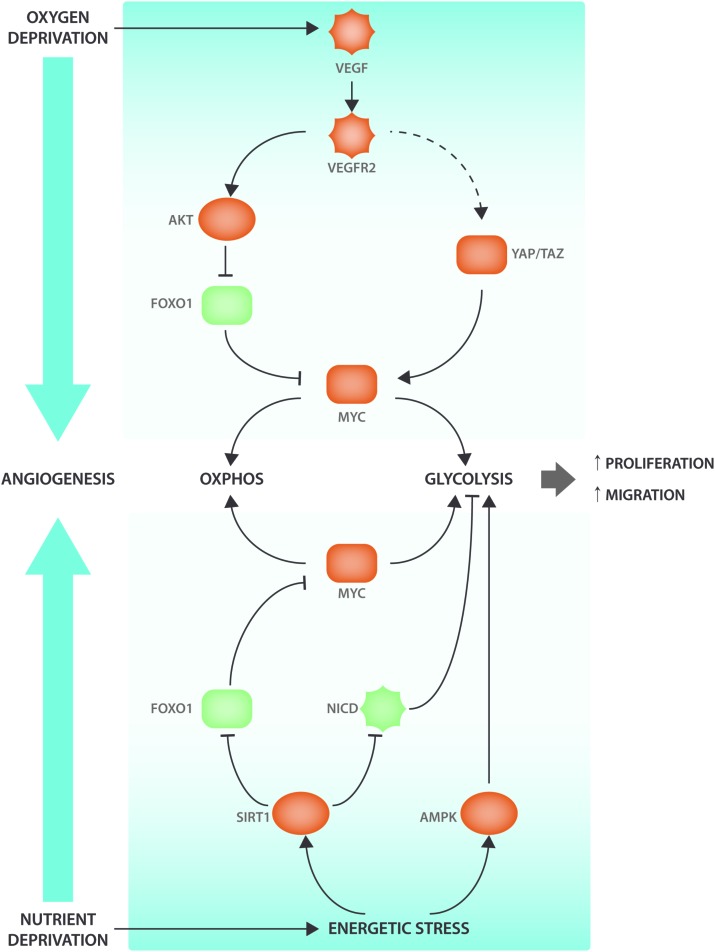
Angiogenic signaling cascades interact with metabolic regulators. VEGF signaling interacts with the ECs’ nutrient sensing apparatus to induce metabolic changes and promote angiogenesis. When oxygen levels decrease, the surrounding tissue will secrete VEGF that will bind to its receptor VEGFR2 present on the ECs. VEGF-induced AKT activation will lead to FOXO1 phosphorylation. When FOXO1 is phosphorylated, it will be excluded from the nucleus and therefore be unable to inhibit MYC transcriptional activity. Moreover, VEGF also leads (via cytoskeletal rearrangements) to YAP/TAZ activation, two transcription factors that have been shown to be involved in metabolic reprogramming via the transcription factor MYC. AMPK and SIRT1 are nutrient sensors that become activated under energetic stress conditions. SIRT1 modulates NICD and FOXO1 activity through deacetylation. AMPK activation enhances EC migration and proliferation by increasing glycolysis. Pro-angiogenic factors are indicated in red and anti-angiogenic factors are indicated in green.

While VEGF-DLL4-Notch is the main signaling hub involved in the control of vessel sprouting many other growth factor and metabolic signaling pathways interact with angiogenic signaling events (**Figure 1**). The NAD^+^ dependent deacetylase SIRT1 nutrient sensor deacetylates and inactivates Notch to control sprouting (Guarani et al., 2011). SIRT1 also deacetylates the Forkhead box O 1 (FOXO1) transcription factor that controls many aspects of tissue growth, maintenance and metabolism and acts as a gatekeeper of EC quiescence by reducing vascular sprouting (Wilhelm et al., 2016). Endothelial FOXO1 is also inactivated by VEGFR2 in a PI3K/AKT dependent manner (Abid et al., 2004). FOXO1 acts through suppressing the expression of MYC, a crucial regulator of growth and metabolism in many cell types, including ECs (Dang, 2013; Wilhelm et al., 2016). MYC expression and activity is also controlled by YAP/TAZ (Kim J. et al., 2017), but it is not clear whether YAP and TAZ mediated control of proliferation and metabolism occurs exclusively via MYC. Intriguingly, it has been shown that YAP/TAZ can also promote EC proliferation by directly binding *cis* elements of metabolic genes (Wang et al., 2017). Another sensor of EC nutrient depletion is AMPK, which promotes angiogenesis under hypoxic conditions (Dagher et al., 2001; Nagata et al., 2003). Thus, main drivers of cellular metabolism interact with VEGF-YAP/TAZ-Notch signaling at many levels to codetermine and modulate sprouting characteristics.

It has recently been shown that ECs change their metabolism during angiogenesis and increase their metabolic activity to meet the specific bioenergetic and anabolic demands that are required for increased migration and proliferation (De Bock et al., 2013a; Eelen et al., 2018). Interestingly, the metabolic switch in ECs not only accompanies the changes in cell phenotype but also plays a critical role in determining cellular behavior during angiogenesis (De Bock et al., 2013b; Schoors et al., 2015). In the following section, we will provide a review of how various metabolic pathways contribute to phenotypic changes during sprouting angiogenesis in healthy conditions as well as how these pathways can be targeted during pathological angiogenesis in cancer.

## EC Metabolism

### Endothelial Cells Are Highly Glycolytic

In many cell types, mitochondria produce the majority of ATP via the oxidative phosphorylation (OXPHOS) of reducing equivalents which are generated in the tricarboxylic acid (TCA) cycle through the catabolism of nutrients. This process requires oxygen and most cells will only switch to glycolytic ATP production under hypoxic conditions. Although they are located next to the bloodstream, and therefore have access to the highest levels of oxygen, ECs predominantly produce ATP via aerobic glycolysis, also termed the Warburg effect. This means that almost all glucose is catabolized into lactate even with ample oxygen availability. Consequently, ECs generate more than 80% of their ATP via glycolysis (Krutzfeldt et al., 1990; Culic et al., 1997; De Bock et al., 2013b) and less than 1% of the pyruvate that is generated by glycolytic breakdown of glucose ends up in the TCA cycle (Krutzfeldt et al., 1990). In fact, blocking pyruvate conversion into lactate by inhibiting lactate dehydrogenase (LDHA), thereby allowing pyruvate entry into the mitochondria, impairs endothelial growth, indicating that recycling of NAD^+^ by LDHA is required to keep EC glycolysis high (Parra-Bonilla et al., 2010). Glycolysis is critical for ECs, and its complete blockade by using 2-deoxy-glucose leads to decreased proliferation and migration and induces cell death (Delgado et al., 2010; Merchan et al., 2010; Schoors et al., 2014).

When compared to other cell types in the body, ECs have high glycolysis and exhibit similar glycolytic rates to many cancer cell lines (De Bock et al., 2013b). These high levels of glycolysis in ECs are maintained through control of several rate limiting steps such as the phosphorylation of glucose to glucose-6-phosphate by hexokinase 2 (HK2) and the conversion of fructose-6-phosphate to fructose-1,6-phosphate by phosphofructokinase 1 (PFK1). In ECs, the activity of PFK1 is controlled by phosphofructokinase-2/fructose-2,6-bisphosphatase 3 (PFKFB3), which produces fructose-2,6-bisphosphate (F2,6P_2_), the main allosteric activator of PFK1 (Van Schaftingen et al., 1982).

#### Why Glycolysis?

Even though glycolytic ATP production occurs at a lower yield when compared to OXPHOS (2 versus 36 mole of ATP per mole of glucose), is less efficient and considered wasteful, several reasons might explain why ECs are highly glycolytic:

First, regulation of glycolytic flux occurs extremely fast (within seconds to minutes) while the response of OXPHOS to increased ATP requirement is at least 100 times slower (Pfeiffer et al., 2001). Glycolysis thus would allow ECs to rapidly adapt their metabolism to the increased energetic demands during proliferation and migration in response to VEGF stimulation and, therefore, to start sprouting immediately.

Second, glycolysis increases the rate of ATP production and can also provide precursors for biomass synthesis (Vander Heiden et al., 2009). This implies that more ATP can be produced during periods of migration where ATP requirements are peaking. Research from the cancer field has shown that production of ATP by glycolysis, rather than OXPHOS, supports cell migration (Yizhak et al., 2014). At the same time, metabolites are generated that can rapidly be shunted into biosynthetic pathways for EC proliferation (Vander Heiden, 2011). For instance, the hexosamine biosynthesis pathway (HBP) uses glutamine, acetyl-CoA and uridine to convert fructose-6-phosphate, a glycolytic intermediate, to glucosamine-6-phosphate and subsequently to uridine-5-diphosphate-*N*-acetylglucosamine (UDP-GlcNAc). UDP-GlcNAc is an important substrate for *O*- and *N*-glycosylation which determines the functionality of numerous proteins including VEGFR2 and Notch (Vaisman et al., 1990; Benedito et al., 2009). The HBP also controls the synthesis of hyaluronan, a critical component of the glycocalyx interface between the endothelium and the vascular lumen (Moretto et al., 2015). Via its dependence on the availability of several nutrients, the HBP potentially acts as a nutrient sensing mechanism that integrates nutrient availability with sprouting behavior. Inhibition of HBP reduces angiogenesis, but the underlying mechanisms still need to be defined (Merchan et al., 2010). Glucose can also leave the glycolytic pathway and enter the pentose phosphate pathway (PPP) to fuel the synthesis of ribose-5-phosphate, which is required for the biosynthesis of nucleotides (Pandolfi et al., 1995). The PPP consists of an oxidative (oxPPP) and non-oxidative branch (non-oxPPP), and inhibition of either of those branches impairs EC viability and migration (Vizan et al., 2009). The flux through the oxPPP is controlled by glucose-6-phosphate dehydrogenase, whose activity is partially controlled by VEGF (Pan et al., 2009). The oxPPP also produces NADPH from NADP^+^ and thus couples nucleotide synthesis to cellular redox status. Glycolytic intermediates can also enter the serine biosynthesis pathway, which in ECs is required for proliferation and survival due to its role in the support of both nucleotide and heme synthesis (Vandekeere et al., 2018) (see below). Taken together, glycolysis will allow ECs to dynamically switch their metabolism when shuffling between the tip and stalk position during sprouting.

Third, because angiogenesis and the restoration of oxygen and nutrient delivery is crucial for survival of the tissue (or even the organism during embryo development) (Carmeliet et al., 1996; Ferrara et al., 1996), proper vascular remodeling has the highest priority. Studies have shown that glycolysis gives cells a competitive advantage when compared to more oxidative cell types when they need to compete for the same glucose (Pfeiffer et al., 2001). Only high glycolytic flux will therefore allow ECs to invade the environment and to receive sufficient glucose for energy production, while the oxidative cells in the microenvironment can exploit alternative sources for ATP production. Glycolysis will also make ECs more resistant to hypoxia as they can use glycolysis anaerobically as long as glucose is available. An alternative way of glycolytic ATP production could be through the breakdown of glycogen during periods when the growing sprouts enter areas where glucose is scarce. Glycogen is an intracellular glucose store and its catabolism to pyruvate yields additional ATP since it does not require glucose uptake followed by hexokinase mediated phosphorylation. Glycogen breakdown might allow ECs to migrate and proliferate when glucose availability is compromised. Along this line, it has been shown that ECs can store glycogen (Amemiya, 1983; Vizan et al., 2009) and levels of GLUT1 are low at the migrating front of the developing retina (Kishimoto et al., 2016). However, whether glycogen breakdown occurs during sprouting and contributes to the metabolic ‘fitness’ of the tip cell, is not known.

Fourth, the preferential utilization of anaerobic ATP production would protect ECs from oxidative stress. By using anaerobic glycolysis, ECs reduce the production of reactive oxygen species (ROS) as a consequence of oxidative metabolism (De Bock et al., 2013a). Future research will be needed to reveal whether quiescent ECs that line the oxygen-rich bloodstream need additional metabolic adaptations to promote NAPDH (and glutathione) production to maintain redox balance.

And last, since providing oxygen and nutrients to the surrounding more oxidative cells is an important role of the vasculature, high glycolysis may allow maximal oxygen diffusion over the endothelial wall.

#### Are Mitochondria Important for ATP Production in ECs?

Endothelial cells rely mainly on glycolysis for ATP production when compared to other cells (De Bock et al., 2013b), and only produce a minor fraction of their ATP via the OXPHOS of reducing equivalents in the mitochondria (Krutzfeldt et al., 1990; Culic et al., 1997; De Bock et al., 2013b). The role of the mitochondrial derived ATP in EC metabolism during sprouting is still not completely understood and is influenced by many factors. For instance, while inhibiting the import of fatty acids (FAs) into the mitochondria does not affect mitochondrial ATP production under normal culturing conditions (Schoors et al., 2015), it does reduce oxygen consumption under conditions where ECs are quiescent and preloaded with oleic acid and rely more on lipid oxidation for ATP production (Kuo et al., 2017). This indicates that under specific conditions, nutrient availability affects the contribution of the mitochondria to ATP production and that ECs might exhibit a Pasteur effect. Moreover, interfering with mitochondrial metabolism does not only alter ATP production but also affects mitochondrial ROS production and cellular redox status, which can modulate EC function. Low mitochondrial ROS levels promote angiogenic signal transduction and migration upon angiogenic stimulation (Chua et al., 1998; Wright et al., 2008; Wang et al., 2011), while higher ROS levels can cause cell damage and death (Wellen and Thompson, 2010; Warren et al., 2014; Vandekeere et al., 2018).

Nonetheless, inhibition of mitochondrial ATP synthesis in ECs via inhibition of ATP synthase does not inhibit endothelial sprouting in a spheroid model (De Bock et al., 2013b). In fact, inhibiting OXPHOS activity might even promote EC migration and sprouting (De Bock et al., 2013b; Longchamp et al., 2018). This increase in migration was caused by an acute activation of the cellular energy sensor AMPK that resulted in a compensatory increase in glycolysis (Longchamp et al., 2018). The inhibition of mitochondrial ATP production might thus have been compensated for by enhanced glycolysis to drive migration. Indeed, inducing mitochondrial dysfunction in osteosarcoma cells enhances glycolysis to maintain NADH recycling, and this sufficed to drive faster migration (Gaude et al., 2018). On the other hand, increasing mitochondrial ATP production and oxygen consumption via supplementing pyruvate does not further promote sprouting nor does it rescue a PFKFB3 knockout driven sprouting defect suggesting that mitochondrial ATP production (from either glucose or FA oxidation) is dispensable during sprouting. Altogether, these data show that ECs tightly control overall energy balance. Although mitochondria do not reach filopodia and lamellipodia during migration, and mitochondrial ATP production occurs too far away from the actin cytoskeleton during sprouting, depleting ATP levels through OXPHOS inhibition results in a metabolic rewiring that promotes glycolysis even at distant sites in the cell and thus drives migration.

### Tip Cells - Compartmentalized Glycolytic ATP Production Drives Migration

**FIGURE 2:**
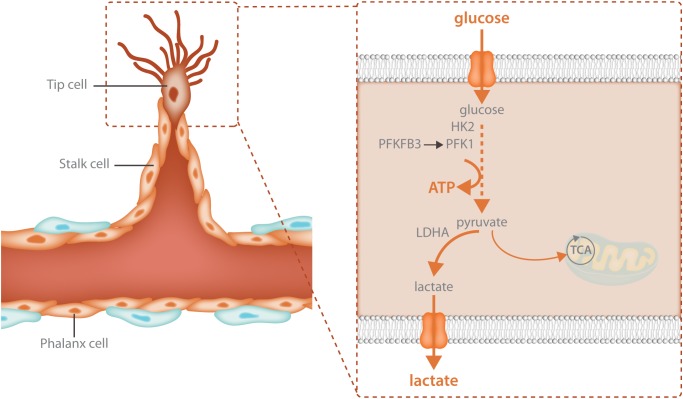
Glycolysis fuels the migrating tip cell. Tip cells are dependent upon glycolytic ATP production and have even higher levels of glycolysis than other endothelial cells. Tip cells increase glycolysis through upregulation of the enzymes that control the rate limiting steps of the reaction: HK2 and PFKFB3. HK2 catalyzes the first reaction of glycolysis, the phosphorylation of glucose to glucose-6-phosphate while PFKFB3 produces fructose-2,6-bisphosphate, the main allosteric activator of PFK1. The majority of the pyruvate that is produced during glycolysis is further broken down into lactate while only 1% ends up in the TCA cycle. High glycolytic flux provides increased local ATP production for the energetically demanding cytoskeletal rearrangements involved in cellular migration.

Glycolysis is particularly crucial for the migrating tip cell. Due to active cytoskeletal rearrangements (Pollard and Borisy, 2003) and the high activity of membrane channels (Schwab et al., 2012; Karlsson et al., 2013) during migration, ATP consumption in the tip cell is extremely high. To meet these increased energetic demands, tip cells upregulate glycolysis above the high baseline levels of glycolysis already found in non-sprouting ECs (**Figure 2**). Different angiogenic growth factors induce glycolytic activation indicating that this process is a crucial component of the angiogenic response. VEGF increases glycolysis by increasing PFKFB3 expression and FGF activates both HK2 as well as PFKFB3 (De Bock et al., 2013b; Yu et al., 2017). VEGF also upregulates GLUT1, the main endothelial glucose transporter (Yeh et al., 2008). The increase in glycolysis upon growth factor stimulation is required for sprouting since endothelial specific knockdown of PFKFB3 as well as HK2 impairs tip cell migration causing vascular defects *in vivo* (De Bock et al., 2013b; Xu et al., 2014; Yu et al., 2017). Thus, activation of glycolysis is required for vessel sprouting.

PFKFB3 and other glycolytic enzymes are mainly located in the perinuclear cytosol of contact inhibited cells but when ECs are sparsely seeded and start to migrate, these enzymes partially relocate to the leading front to support the ATP-consuming process of cytoskeletal remodeling. Indeed, lamellipodia and filopodia contain a meshwork of polymerized actin where high levels of ATP are found. Knockdown of PFKFB3 reduced lamellipodial ATP levels, indicating that they are derived from glycolysis (De Bock et al., 2013b). In addition, PFKFB3 also immunoprecipitated with β-actin and was more abundant in the F-actin fraction, the filamentous form found in motile lamellipodia and filopodia of migrating ECs (De Bock et al., 2013b). The compartmentalization of glycolytic enzymes and their binding to actin has been documented in other cell types (Schmitt and An, 2017), and has several implications: migration speed and directionality are driven by local ATP production (van Horssen et al., 2009). In this regard, knockdown of PFKFB3 reduces migration speed as well as directionality. F-actin also provides docking sites for other glycolytic enzymes which stabilizes them, increases their activity and allows other enzymes to piggy-back (Real-Hohn et al., 2010; Araiza-Olivera et al., 2013). By lining up several glycolytic enzymes in a highly organized fashion using the actin as a scaffold, a so called ‘metabolon’ is formed. In these metabolons, metabolites are channeled, which indicates that the product of one enzyme is immediately transferred to the next enzyme, which enhances metabolic efficiency and further increases the flux through a specific pathway (Miura et al., 2013). The importance of glycolytic compartmentalization is underscored by the observation that flies which lack the ability to compartmentalize aldolase to the actin, results into the inability to fly, even when all enzymes are present (Wojtas et al., 1997).

The signals that control glycolytic enzyme localization in ECs are not known. In mammary epithelial cells, Rac/cdc42 dependent cytoskeletal rearrangements induced by PI3K signaling mobilize the glycolytic enzyme aldolase from the F-actin to control glycolysis (Hu et al., 2016). Along with cytoskeletal tethering, PFKFB3 and many other glycolytic enzymes can relocate to the nucleus. There, PFKFB3 produces F2,6P_2_, which enhances cyclin-dependent kinase-mediated phosphorylation of p27^kip1^ (a potent inhibitor of Cdk and G_1_-to-S cell cycle phase transition) thereby promoting its proteasomal degradation. This results in increased proliferation, independent of increased glycolysis (Yalcin et al., 2009). Acetylation of PFKFB3 in HeLa cells leads to its cytoplasmic accumulation where it contributes to increasing glycolysis (Li et al., 2018). Interestingly, deacetylation of PFKFB3 seems to be regulated by SIRT1 (Li et al., 2018). Accordingly, HK2 localizes both in the cytoplasm as well as at the mitochondrial membrane, and its multifunctional role can be dependent or independent of its kinase activity (Pastorino and Hoek, 2008; Snaebjornsson and Schulze, 2018). Knockdown of pyruvate kinase M2 (PKM2) in ECs reduces spheroid sprouting (Boeckel et al., 2016), but it remains to be elucidated whether, in agreement with cancer cells (Yang et al., 2014), PKM2 can also be present in the nucleus under some conditions to control gene expression and proliferation independent of its pyruvate kinase activity. As many glycolytic enzymes have been shown to exert non-canonical functions, which are dependent on their location, further studies on the exact localization and regulation of glycolytic enzymes will provide valuable insight into the compartmented organization of EC metabolism and how this affects sprouting.

Besides controlling tip cell migration, glycolysis also determines the ability of ECs to take the tip cell position. Mice that lack PFKFB3 or HK2 in ECs have a lower number of tip cells and the tip cells that are present have fewer and shorter filopodia (De Bock et al., 2013b; Yu et al., 2017). PFKFB3 overexpression promotes tip cell contribution even in cells that have been genetically instructed to exhibit a stalk cell phenotype via overexpressing the Notch intracellular domain (NICD), which leads to activation of the Notch transcriptional program. These data might have interesting implications that require further testing because acquiring the tip cell position by tip cell overtaking is considered to occur spontaneously (Arima et al., 2011; Boas and Merks, 2015). Therefore, increasing the potential for fast migration might increase the likelihood that a particular cell ends up at the tip position. In that case, tip cell competition is in fact a ‘running’ race for the tip that will be won by the fastest one, where speed is determined by the kinetics of ATP requiring processes such as actin cytoskeletal rearrangements and VE-Cadherin recycling (Cruys et al., 2016). Alternatively, since PFKFB3 positive ECs have more filopodia and lamellipodia compared to PFKFB3 knockout ECs, high glycolysis can promote the ability of ECs to execute the tip cell function once they have acquired the tip cell position.

### Stalk Cells - When Mitochondria Contribute to Biomass Synthesis

**FIGURE 3:**
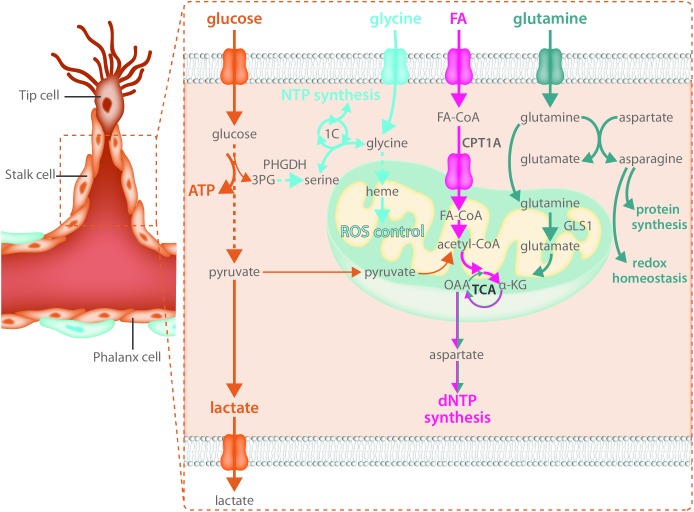
Stalk cells use fatty acid oxidation and amino acid metabolism for biomass synthesis. Like all endothelial cells, stalk cells rely heavily on glycolysis for ATP production. Fatty acid oxidation and amino acid metabolism are required to produce the macromolecules necessary for stalk cell proliferation. Carbon derived from fatty acid oxidation and glutamine catabolism contribute to nucleotide synthesis while glutamine and asparagine catabolism contribute to both protein synthesis and maintenance of redox homeostasis. The intracellular serine synthesis pathway links glucose metabolism with amino acid metabolism in stalk cells for the production of NTPs as well as heme synthesis, which maintains mitochondrial function and controls ROS production.

Endothelial cells rely heavily on glycolytic ATP production as an energy source in not only the tip cell but in stalk cells as well (**Figure 3**). Reducing glycolysis in ECs leads to vascular defects by impairing tip cell function as well as stalk cell proliferation (Yu et al., 2017). While the role of mitochondria in the migrating tip cell requires more investigation, it has been shown that they critically contribute to EC metabolism in the stalk cell by acting as a biosynthetic hub for cellular proliferation. The TCA cycle is an important contributor to the generation of many metabolic intermediates for the *de novo* synthesis of nucleotides, proteins and lipids in many proliferating cell types (Pavlova and Thompson, 2016). Besides glucose, long chain FAs can produce acetyl-CoA upon beta-oxidation in the mitochondria. Transport of FAs into the mitochondria is controlled by carnitine palmitoyl transferase 1 alpha (CPT1A), the rate limiting enzyme of fat oxidation (FAO) (Eaton, 2002). In ECs, FA derived carbons are incorporated into many TCA cycle intermediates (Schoors et al., 2015) and loss of CPT1A causes endothelial sprouting defects (Schoors et al., 2015). This was due to reduced biomass synthesis, particularly reduced deoxyribonucleotide (dNTP) synthesis (Schoors et al., 2015) (**Figure 3**). FA derived acetyl-CoA was found to be the major carbon source for TCA cycle intermediates including citrate, α-ketoglutarate (αKG), glutamate, and importantly aspartate which is an essential carbon source for dNTP synthesis (Schoors et al., 2015). CPT1A knockdown in cultured ECs severely blunted the contribution of FA derived carbon to dNTPs and reduced totals levels of dNTPs (Schoors et al., 2015). The reduced dNTP synthesis resulted in decreased proliferation of ECs *in vitro* as well as reduced EC proliferation in the developing retinal vascular network, resulting in decreased sprout length as well as branching complexity *in vivo* (Schoors et al., 2015). However, CPT1A was not required for migration and did not change tip cell number nor the amount of filopodia (Schoors et al., 2015). The contribution of FA oxidation to non-lipid biomass seems to be a feature that is restricted to ECs as recent evidence indicates that in many other cell types, FAO does not provide carbon to non-lipid biomass (Hosios et al., 2016).

Along with a reliance on FAO for biomass production, ECs use the non-essential amino acid glutamine to sustain proliferation and macromolecular biosynthesis (Huang et al., 2017; Kim B. et al., 2017). Proliferating ECs consume high amounts of glutamine, more than other amino acids in the media (Krutzfeldt et al., 1990; Huang et al., 2017; Kim B. et al., 2017). Once inside the cell, glutamine is metabolized to glutamate by glutaminase (GLS). Glutamate is then converted to αKG by glutamate dehydrogenase (GDH) or transaminases and contributes to protein and nucleotide synthesis by entering the TCA cycle (**Figure 3**). αKG-derived oxalacetate may exit the TCA cycle and be converted to aspartate that will be further used for protein and/or nucleotide biosynthesis. Moreover, αKG could be metabolized to citrate via reductive carboxylation and contribute to FA synthesis. Glutamine depletion, or genetic inactivation of *GLS1*, blunts EC proliferation due to depletion of TCA metabolites and a subsequent decrease in macromolecular biosynthesis (Huang et al., 2017; Kim B. et al., 2017). However, it is unclear whether glutamine also contributes to ATP production in proliferative ECs. Kim B. et al. (2017) reported a 50% reduction in energy charge in glutamine depleted and siGLS1 cells, whereas Huang et al. (2017) showed that glutamine deprivation does not have an effect on ATP production in ECs. When glutamine is available, EC synthesize asparagine by converting glutamine-derived nitrogen (and aspartate) to asparagine via asparagine synthase (*ASNS*) to support cell growth, mTORC activation and protein synthesis as well as to reduce endoplasmic reticulum (ER) stress (Huang et al., 2017) (**Figure 3**). Accordingly, increased ER stress induced by glutamine depletion was alleviated when αKG and asparagine were provided (Huang et al., 2017). Moreover, reduced proliferation in glutamine-deprived ECs could be restored by αKG and asparagine supplementation (Huang et al., 2017), indicating that both carbon and nitrogen sources are important for EC proliferation. In addition, glutamine-deprived ECs activate macropinocytosis in order to take up asparagine (and other nutrients) from the extracellular milieu (Kim B. et al., 2017). However, whether macropinocytosis also ensures endothelial asparagine uptake *in vivo* is not known and further studies using genetic inactivation of both pathways are needed to validate the *in vitro* data.

The role of other amino acids in EC biology needs to be further characterized. In this regard, recent data show that VEGF increases the expression of the glycine transporter 1 and enhances intracellular glycine availability (Guo et al., 2017). This promotes tube formation *in vitro* and perfusion of the ischemic hindlimb *in vivo* by improving mitochondrial coupling, reducing ROS, and promoting NO synthesis. At the same time, ECs are also dependent on intracellular glycine synthesis via the serine biosynthesis pathway. Indeed, VEGF increases the expression of enzymes involved in serine biosynthesis, and intracellular glycine levels cannot be maintained in the absence of phosphoglycerate dehydrogenase (PHGDH), a crucial enzyme within the serine biosynthesis pathway. Interestingly, knockdown of PHGDH has recently been reported to critically inhibit angiogenesis *in vitro* and *in vivo* (Vandekeere et al., 2018). *In vivo*, endothelial specific PHGDH knockout mice exhibit decreased EC proliferation as well as increased vessel regression due to lower EC survival and pups die around P9 while *in vitro*, PHGDH knockdown in ECs impairs vessel sprouting from a spheroid due to decreased proliferation. These defects were not only dependent on decreased nucleotide synthesis leading to decreased proliferation but were also – in contrast to many other cell types – dependent on reduced heme synthesis (**Figure 3**). Since heme is crucial to maintain the function of the electron transport chain, impaired heme synthesis increased oxidative stress thereby reducing EC survival (Vandekeere et al., 2018). Amino acids might also control EC tip cell behavior through yet undefined mechanisms. GLS1 inhibition affects EC migration, reduces the number of tip cell in the developing retina *in vivo* and impairs the ability to obtain the tip cell position in competition assays, indicating that amino acid metabolism also contributes to tip cell behavior (Huang et al., 2017). In support of this, microarray data from retinal tip cells showed enrichment of GLS2, though the role of GLS2 in sprouting still needs to be investigated (Strasser et al., 2010).

### Phalanx

As mentioned before, when a new blood vessel is fully formed, ECs stop migrating and proliferating, and become quiescent. These phalanx cells form a tight monolayer of cobblestone-like cells which resembles the phalanx formation of ancient Greek soldiers (Mazzone et al., 2009). Quiescent phalanx cells are characterized by an exit from the cell cycle, entering a state of reversible cell cycle arrest in G0/G1, a condition in which they can stay for years. Although termed ‘quiescent,’ phalanx cells need to actively secrete a glycocalyx layer to ensure optimal perfusion, protect themselves from the harmful oxygen rich environment by maintaining redox status, provide optimal barrier function, and maintain vasoregulation (Pries et al., 2000; Kops et al., 2002; Potente et al., 2011). In addition, while it is plausible that they need to adapt their own metabolism to this multifaceted role, at the same time, they need to provide optimal oxygen and nutrient availability to the microenvironment. However, little is known about how ECs change their metabolism during transition to the quiescent state. Moreover, how this quiescent state is metabolically maintained, and how metabolic interaction with the microenvironment that the blood vessel serves is controlled, is not known.

One of the factors that induces quiescence is shear stress, induced by the force that laminar flow exerts on ECs (Lee et al., 2012). Krüpple-like factor 2 (KLF2) is a transcription factor that is induced by shear stress and that orchestrates a network of genes that control EC quiescence (Doddaballapur et al., 2015). Shear stress reduces the expression of the glycolytic enzymes HK2, PFK1 as well as PFKFB3 through the direct binding of KLF2 to its gene promoter (Doddaballapur et al., 2015). Overexpression of PFKFB3 partially blunts the KLF2 mediated reduction in sprouting, indicating that the ability of KLF2 to induce quiescence is co-determined by its ability to repress glycolysis. Knockdown of PFKFB3 in ECs is indeed sufficient to induce a quiescent phenotype (Schoors et al., 2014). Shear stress also reduces oxygen consumption *in vitro* but this is highly dependent on the oxygen tension in which cells are maintained (Jones et al., 2008), so it is not clear yet whether quiescent ECs also respire less *in vivo*. Opposite to this, disturbed flow activates glycolysis and downregulates mitochondrial respiration, and targeting this glycolytic switch reduces inflammation (Wu et al., 2017). Besides KLF2, quiescent ECs show high levels of active FOXO1, which couples reduced EC proliferation with reduced glycolysis as well as mitochondrial activity. Mechanistically, FOXO1 decelerates EC metabolism and reduces metabolic rate by repressing MYC signaling (see above) (Wilhelm et al., 2016). As mentioned above, FOXO1 is inhibited by PI3K/AKT mediated phosphorylation which leads to its nuclear exclusion. FOXO1 is also inhibited following SIRT1 mediated deacetylation (Abid et al., 2004; Wilhelm et al., 2016). The endothelium is extremely sensitive to alterations in FOXO1 levels. During development, FOXO1 deficiency leads to EC hyper-proliferation, hyperplasia and vessel enlargement, while FOXO1 overexpression results in defective blood vessel development and hypobranching (Wilhelm et al., 2016). Moreover, in phalanx ECs intracrine VEGF signaling controls viability by preventing FOXO1 overactivation and subsequent suppression of glycolysis, mitochondrial respiration and FA synthesis (Domigan et al., 2015).

Endothelial cells rewire their metabolism by decreasing glycolysis and mitochondrial activity when becoming quiescent (Doddaballapur et al., 2015; Patella et al., 2015; Wilhelm et al., 2016) while at the same time increasing FAO (Patella et al., 2015). When fully assembled into a three dimensional network, ECs use FAO to replenish TCA cycle intermediates to sustain ATP production (Patella et al., 2015). Inhibition of CPT1A lowered ATP levels and glucose consumption which could almost completely be restored by forcing glucose entry into the TCA cycle. Interestingly, inhibition of FAO increased EC permeability *in vitro* and vessel leakiness *in vivo*, and this was dependent on the contribution of FAO to OXPHOS, as inhibition of oligomycin had similar effects. However, the reduction in ATP levels was only around 10%, indicating that other mechanisms might potentially contribute to the ability of FAO to maintain barrier function. In this regard, the production of FAO derived NADPH, which is key to counteract oxidative stress in tumor cells (Pike et al., 2011), might be an additional mechanism through which FAO maintains vascular health. In addition, recent reports have shown that ECs also have the capacity to store lipids in lipid droplets (Kuo et al., 2017). These lipids can be used for the production of ATP via FAO or potentially NADPH. It is not yet clear to what extent the formation of lipid droplets in ECs is relevant to their energy homeostasis, although a role in the regulation of energetic substrate provision to parenchymal tissue has been proposed (Dagher et al., 2001; Ibrahim and Arany, 2017; Kuo et al., 2017). Recent *in vivo* visualization of FA flux over the endothelium however failed to show lipid accumulation inside ECs (He et al., 2018), indicating that more research is needed to identify how quiescent ECs metabolically interact with their microenvironment.

Quiescent ECs expressing Notch1 and NICD (indicating active Notch1 signaling) have been observed at least in heart ECs (Jabs et al., 2018). In addition, activation of Notch signaling is one of the mechanisms through which ECs become contact inhibited when grown until confluence (Noseda et al., 2004; Rostama et al., 2015). Chronic treatment with anti-DLL4 antibodies induces vascular neoplasms due to pathological activation of ECs in mice (Yan et al., 2010) and leads to congestive heart failure in humans in phase I clinical studies (Smith et al., 2014; Chiorean et al., 2015). These data suggest that Notch has an important role in the maintenance of vascular quiescence. Whether it does so via altering EC metabolism still needs to be explored, however, contact inhibited cells have lower glycolysis and NICD overexpression in ECs reduces PFKFB3 and glycolysis (Schoors et al., 2014). Moreover, Notch engages in the metabolic crosstalk between the endothelium and the periphery by controlling vascular lipolysis and transendothelial transport of FAs into the heart by transcriptionally controlling key genes in these processes (Jabs et al., 2018). In addition, global inhibition of Notch improves insulin sensitivity, but it is not known whether this effect is (co-)mediated by the vasculature (Pajvani et al., 2011).

### Nutrient Depletion and Angiogenesis

Although mounting evidence indicates that sprouting critically depends on glycolysis, FAO, and glutaminolysis, it is paradoxical that angiogenesis is in fact driven by tissue nutrient deprivation. Nascent sprouts must be instructed to grow, divide, and migrate by the nutrient limited conditions of the host tissue. Recent work has shed light on how nutrient deprivation can initiate angiogenesis. The NAD^+^ dependent deacetylase SIRT1 is activated by increases in NAD^+^ levels during nutrient deprivation or cellular energy shortage. Upon nutrient restriction, deacetylation of NICD by SIRT1 lowers NICD stability and desensitizes ECs to Notch activation by dampening the Notch response. Accordingly, loss of EC SIRT1 imposes a non-sprouting, stalk cell phenotype (Guarani et al., 2011). In addition to NICD, SIRT1 also deacetylates FOXO1, thereby limiting its antiangiogenic activity (see below) (Wilhelm et al., 2016). The fact that nutrient restriction can directly alter angiogenic behavior of ECs is underscored by the observation that dietary restriction by reducing caloric intake promotes angiogenesis in the ischemic hindlimb (Kondo et al., 2009). In fact, removing the sulfur amino acids (SAA) methionine and cysteine from the diet phenocopies many aspects of dietary restriction (Orentreich et al., 1993). Depleting SAA alone increases vascular density in skeletal muscle in a VEGF dependent manner and improves neoangiogenesis after femoral artery ligation (Longchamp et al., 2018). SAA restriction of cultured ECs increased angiogenic capacity in a VEGF and SIRT1 dependent manner. Mechanistically, this was due to an increase in the production of hydrogen sulfide (H_2_S) by endothelial cystathionine-gamma-lyase (CGL) during SAA restriction. H_2_S inhibits complex I and IV of the electron transport chain thereby reducing OXPHOS dependent oxygen consumption. This evoked an AMPK-dependent compensatory increase in glycolytic ATP production as well as increased flux through the PPP (Longchamp et al., 2018) each of which are required for angiogenesis (Vizan et al., 2009; De Bock et al., 2013b). These data shed light on how the balance between the absence of specific nutrients and the presence of others can drive angiogenesis.

## Tumor ECs - Targeting Endothelial Metabolism for Anti-angiogenesis or Vessel Normalization

**FIGURE 4:**
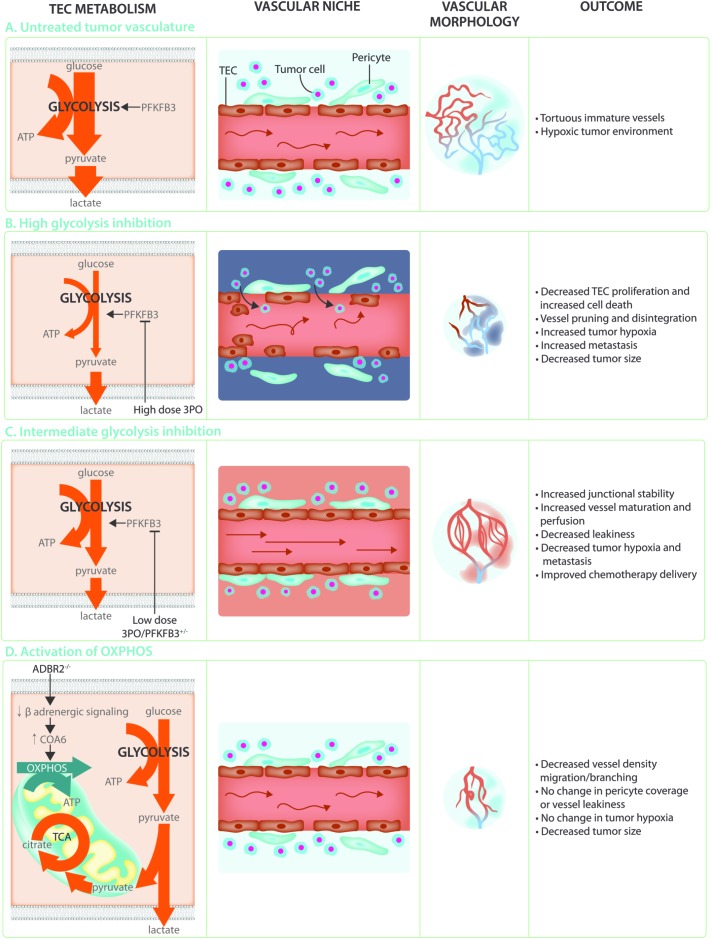
Targeting TEC metabolism as anti-cancer treatment. **(A)** The hypoxic and acidic tumor microenvironment leads to hyperactivation of the tumor endothelium. This leads to TECs that are hyperglycolytic in comparison to normal endothelial cells, weakly connected due to decreased VE-Cadherin, and poorly covered by pericytes. These abnormal characteristics lead to poor perfusion and a worsening of the hypoxic and acidic tumor microenvironment establishing a loop of non-productive angiogenesis creating tortuous and immature tumor vasculature. **(B)** Targeting the hyperglycolytic TEC metabolism by high dose 3PO treatment decreases TEC proliferation and increases endothelial cell death. While this leads to increased vessel pruning, disintegration, and decreased tumor size, it also increases tumor hypoxia and metastasis. **(C)** Targeting TEC glycolysis by using low dose 3PO treatment, or in PFKFB3+/– mice, induces an intermediate brake on TEC metabolism leading to vessel normalization. Increased junctional stability due to decreased endocytosis of the junctional molecule VE-Cadherin allows for decreased vascular leakiness and increased vessel maturation. Normalization of the tumor vasculature improves tumor perfusion, hypoxia, and chemotherapy delivery while reducing metastasis. **(D)** While TECs rely mainly on glycolysis for ATP production, increasing OXPHOS through decreasing β-adrenergic signaling decreases migration and proliferation thereby preventing the angiogenic switch. This does not improve vessel maturation nor influence tumor hypoxia but reduces tumor growth.

Angiogenesis is crucial to support tumor growth and malignancy (Folkman, 1971; Hanahan and Folkman, 1996). Indeed, rapidly dividing cancer cells have an increased requirement for nutrients and oxygen to support biomass synthesis and ATP production, and at the same time their own rapid growth causes hypoxia. This leads to an uncontrolled and relentless production of pro-angiogenic factors which induces hyperactive and abnormal vessel growth. Tumor vessels are abnormal in both structure and function, being characterized as tortuous, morphologically heterogeneous, and disorganized. Tumor ECs (TECs) are poorly interconnected due to lower levels of the junctional molecule VE-Cadherin, have lost polarity, are poorly covered with pericytes and often leave gaps which reduces barrier function and allows tumor cells to escape and metastasize to distant organs (Jain, 2005; Carmeliet and Jain, 2011; Cantelmo et al., 2016) (**Figure 4A**). In addition, these TECs are irregular and no longer form a tight monolayer but become stacked and protrude into the vessels lumen. These abnormalities lead to perturbed perfusion of the tumor and worsening of the hypoxic, acidic and nutrient deprived conditions within the tumor microenvironment. This results in the secretion of even more angiogenic growth factors thereby establishing a loop of non-productive angiogenesis further promoting vessel abnormalization and tumor malignancy. Because the abnormality of the tumor blood vessels contributes to the severity of tumor malignancy, blood vessel normalization has become a promising strategy for anti-angiogenic cancer therapy. The initially proposed concept of anti-angiogenic treatment, which was intended to induce vessel pruning and starve the tumor to death, has met with little success due to development of resistance as well as an intensification of the hypoxic/acidic microenvironment which can promote metastatic spreading (Ebos et al., 2009; Paez-Ribes et al., 2009). In contrast, promoting vascular normalization would improve tumor perfusion, reduce hypoxia, prevent invasion/metastasis, and increase delivery and efficacy of chemotherapy (Jain, 2001; Carmeliet and Jain, 2011; Goel et al., 2012).

Over the last few years, it has become clear that TECs have an altered metabolic profile (Carmeliet and Jain, 2011; Cantelmo et al., 2016). Indeed, TECs show an even higher reliance on glycolysis (**Figure 4A**) when compared to normal proliferating ECs and have an increased expression of almost all glycolytic enzymes, including GLUT1, at the transcriptional level as well as increased abundance of glycolytic metabolites (Cantelmo et al., 2016; Jayaraman et al., 2018). Lowering glycolysis by low dose pharmacological PFKFB3 inhibition using 3-(3-pyridinyl)-1-(4-pyridinyl)-2-propen-1-one (3PO) or endothelial PFKFB3 haplodeficiency did not affect tumor growth but normalized tumor vessels leading to lower cancer cell invasion, intravasation and metastasis (Cantelmo et al., 2016). Moreover, these tumors had more mature vessels and showed enhanced vessel perfusion which improved tumor oxygenation. This lead to improved delivery of chemotherapy into the tumor and increased chemotherapeutic efficacy due to higher oxygen availability (Cantelmo et al., 2016) (**Figure 4C**). Mechanistically, PFKFB3 inhibition reduced the endocytosis of VE-Cadherin, thereby tightening the vascular barrier and rendered pericytes more quiescent and adhesive. The effect of PFKFB3 inhibition on the tumor vasculature is dependent though on the degree of inhibition. Tumors implanted in mice with complete PFKFB3 deletion grow more slowly due to reduced tumor perfusion (Xu et al., 2014). Similarly, high doses of the PFKFB3 inhibitor 3PO lead to tumor vessel disintegration. The increase in vascular leakiness that accompanies tumor vessel disintegration promoted tumor cell metastasis (Conradi et al., 2017) (**Figure 4B**). It seems that the balance between vessel pruning and vessel normalization depends on how big the brake on endothelial metabolism is. Indeed, under the metabolically stressful and hypoxic conditions of the microenvironment, complete high dose PFKFB3 inhibition induced TEC apoptosis (Cantelmo et al., 2016). PFKFB3 knockdown also induced apoptosis under hypoxic conditions *in vitro* (Xu et al., 2014). Moreover, ECs infected with Kaposi‘s sarcoma associated herpesvirus which leads to endothelial tumor formation, are more sensitive to glycolytic inhibition associated apoptosis (Delgado et al., 2010). Taken together, targeting endothelial glycolysis can offer an attractive therapeutic opportunity, but caution is warranted since its effects are highly dose-dependent. This notion is particularly relevant in the cancer setting, where clinical trials for glycolytic inhibitors for anti-cancer treatment are currently ongoing, and where effects on the vasculature might co-determine long-term treatment outcome for the patient.

It is not clear yet which factors control the activation of glycolysis in the tumor. Although it has previously been shown that VEGF secreted from hypoxic glioma tumor cells can increase GLUT1 expression in ECs (Yeh et al., 2008), several other factors that are present in the tumor microenvironment such as cytokines, hypoxia and estrogen (Yizhak et al., 2014; Cantelmo et al., 2016; Trenti et al., 2017) can also increase EC PFKFB3 expression. Interestingly, the activated glycolytic transcriptional pattern and increased glycolytic flux can be maintained upon culturing (Cantelmo et al., 2016). It is therefore unlikely that enhanced glycolysis is solely caused by acute environmental conditions within the tumor microenvironment but that other mechanisms including epigenetic modifications (potentially induced by the tumor microenvironment) co-determine TEC glycolysis. Also, recent single cell data obtained from TECs isolated from tumors treated with anti-DLL4 (which inhibits Notch signaling and results in an increased, non-functional vasculature) and/or VEGF inhibition (which reduces tumor vessel density) showed that glycolytic genes were amongst the most activated ones in tip cell-like TECs upon both anti-angiogenic treatments (Zhao et al., 2018). This is remarkable, since reducing VEGF signaling would favor reduced glycolysis. Nonetheless, given the concomitant increase in the expression of hypoxia genes induced by both treatments, it is possible that hypoxia contributes to metabolic regulation of ECs in the tumor. As it is known that endothelial hypoxia signaling mediates the tumor vascular phenotype (Branco-Price et al., 2012), it will be exciting to explore the *in vivo* behavior of highly glycolytic, tip cell-like TECs and how they contribute to anti-angiogenic resistance.

Besides glycolysis, many other metabolic pathways such as the PPP, and the serine biosynthesis pathway are transcriptionally deregulated in TECS (Cantelmo et al., 2016). In addition, culturing ECs in tumor cell derived conditioned medium revealed significant changes in their metabolite profile that were dependent on the type of cancer cell (Jayaraman et al., 2018). Metabolite pathway analysis showed activated glycolysis and purine metabolism as well as FAO which was underscored by a pronounced increase in the levels of acetyl carnitine. The hyperproliferative nature of TECs might require active mitochondria for biomass synthesis and it cannot be excluded that in a competitive cancer setting, where glycolysis is already maximized, mitochondrial ATP synthesis is required for angiogenesis. Indeed, it has been shown that treatment of proliferating ECs with Embelin, a weak mitochondrial uncoupler, causes a reduction in OXPHOS which leads to reduced tumor growth and decreased microvessel density in murine tumor models (Coutelle et al., 2014). Conversely, a recent report indicates that inducing a shift to oxidative metabolism through inhibition of adrenergic signaling in ECs, can prevent the angiogenic switch in a mouse model of prostate cancer leading to decreased tumor growth (Zahalka et al., 2017). Indeed, EC specific knockout of the β_2_-adrenergic receptor (ADBR2) increased the expression of cytochrome C oxidase assembly factor 6 (COA6), leading to an increase in OXPHOS activity. This was supported by increased glucose uptake and an increased contribution of glucose and glutamine oxidation to the TCA cycle without decreasing intracellular lactate. Interestingly, this increase in OXPHOS lead to decreased EC migration and proliferation, despite increased ATP levels (**Figure 4D**). This data indicates that increasing OXPHOS in TECs may directly alter EC migratory and proliferative capacity independent of levels of glycolysis (Zahalka et al., 2017). While the differences between these reports remain to be reconciled, they open up the possibility of pursuing non-glycolytic targets of TEC metabolism as cancer therapies.

TECs are part of a complex tumor microenvironment and are surrounded by not only the malignant cancer cells but also tumor associated macrophages (TAMs), fibroblasts and other stromal cells. The specific context of the tumor microenvironment imposes great metabolic challenges: the uncontrolled and rapid proliferation of cancer cells rapidly creates a hypoxic environment which is exacerbated by the abnormal characteristics of the tumor vasculature. This hypoxic response enhances glycolytic flux in tumor cells leading to a highly acidic microenvironment caused by the production of high levels of lactate (Cairns et al., 2011; Vander Heiden, 2011; Harjes et al., 2012). Lactate can be taken up by TECs through monocarboxylate transporter 1 (MCT1) which promotes angiogenesis. This occurs through increased VEGFR2 levels following the stabilization of hypoxia inducible factor 1 (HIF1) in an αKG and ROS dependent fashion rendering them more responsive to the pro-angiogenic action of VEGF (Vegran et al., 2011; Sonveaux et al., 2012). Incubation of ECs with conditioned medium from glioblastoma tumor cells increases MCT1 expression (Miranda-Goncalves et al., 2017). Moreover, lactate increases PI3K/AKT signaling downstream of angiogenic receptor activation due to increased production of pro-angiogenic factors (Ruan and Kazlauskas, 2013). Increased lactate levels in the hypoxic tumor will thus further tip the balance in favor of vessel abnormalization. *In vivo*, inhibiting lactate transport through MCT1 reduces tumor angiogenesis (Sonveaux et al., 2012). Also, upon exposure to conditioned medium from cancer cells, ECs increase expression of GLUT1 and metabolically prepare for increased angiogenic activity (Yeh et al., 2008). High succinate concentrations in the tumor microenvironment also promote glucose uptake by TECs but it is not clear whether this is via metabolic effects, HIF stabilization or via activation of the succinate receptor GPR91 (Garrigue et al., 2017).

Nutrient limitation in the tumor microenvironment provides an additional metabolic challenge in which different cell types need to compete for nutrients to support biomass generation, bioenergetic needs, as well as effector functions (Lyssiotis and Kimmelman, 2017). For instance, TAMs compete with TECs for the limited glucose in the tumor microenvironment, and stimulating glucose metabolism in TAMs induces vessel normalization (Wenes et al., 2016). The hyperglycolytic TAMs lower glucose availability for TECs so that the latter are subsequently forced toward quiescence and a more normalized phenotype. These glucose starved TECs have tighter VE-Cadherin positive junctions which, like 3PO treatment, increased perfusion, reduced tumor hypoxia and prevented metastasis. Although the metabolic crosstalk and competition between TECs and other cells in the tumor microenvironment remain largely unexplored, due to the unique and extreme conditions within tumor microenvironment, targeting this crosstalk offers windows for therapeutic opportunities. The metabolic cross talk between these cell types is therefore an intriguing topic for exploration.

## Future Perspectives

The role of EC metabolism in vessel sprouting has received considerable attention over the last few years. While our knowledge on how metabolism and angiogenesis interact is growing, only a few pathways have been characterized to date. Undoubtedly, extending our insight into the role of other pathways will offer tremendous insight into basic mechanisms of sprouting and non-sprouting angiogenesis. Furthermore, since metabolism drives angiogenesis, understanding the metabolic differences between healthy and diseased ECs might offer novel treatment opportunities for many diseases such as cancer but also for regenerative purposes. In addition, EC metabolism research has exclusively been performed under *in vitro* conditions and/or using preclinical mouse models. It still remains to be confirmed whether ECs have similar metabolic characteristics in humans and whether those can be exploited for therapy. Another exciting outstanding question is whether ECs alter their metabolism dependent upon the microenvironment in which they reside and the nutrients they have available. This will provide further insight into how they interact with their microenvironment. In this regard, the development of genetic tools that allow tissue restricted endothelial gene regulation will become crucial to overcome limitations of currently available models.

## Author Contributions

All authors listed have made a substantial, direct and intellectual contribution to the work, and approved it for publication.

## Conflict of Interest Statement

The authors declare that the research was conducted in the absence of any commercial or financial relationships that could be construed as a potential conflict of interest.
